# Flavonoids as an effective sensitizer for anti-cancer therapy: insights into multi-faceted mechanisms and applicability towards individualized patient profiles

**DOI:** 10.1007/s13167-021-00242-5

**Published:** 2021-05-17

**Authors:** Alena Liskova, Marek Samec, Lenka Koklesova, Aranka Brockmueller, Kevin Zhai, Basma Abdellatif, Manaal Siddiqui, Kamil Biringer, Erik Kudela, Martin Pec, Laura Kate Gadanec, Miroslava Šudomová, Sherif T. S. Hassan, Anthony Zulli, Mehdi Shakibaei, Frank A. Giordano, Dietrich Büsselberg, Olga Golubnitschaja, Peter Kubatka

**Affiliations:** 1 Department of Obstetrics and Gynecology, Jessenius Faculty of Medicine, Comenius University in Bratislava, 03601 Martin, Slovakia; 2 Musculoskeletal Research Group and Tumor Biology, Chair of Vegetative Anatomy, Institute of Anatomy, Faculty of Medicine, Ludwig-Maximilian-University Munich, 80336 Munich, Germany; 3 Weill Cornell Medicine-Qatar, Education City, Qatar Foundation, 24144 Doha, Qatar; 4 Department of Medical Biology, Jessenius Faculty of Medicine, Comenius University in Bratislava, 03601 Martin, Slovakia; 5 Institute for Health and Sport, Victoria University, Melbourne, 3030 Australia; 6 Museum of Literature in Moravia, Klášter 1, 66461 Rajhrad, Czech Republic; 7 Department of Applied Ecology, Faculty of Environmental Sciences, Czech University of Life Sciences Prague, Kamýcká 129, 16500 Prague, Czech Republic; 8 Department of Radiation Oncology, University Hospital Bonn, Rheinische Friedrich-Wilhelms-Universität Bonn, Bonn, Germany; 9 Predictive, Preventive and Personalised (3P) Medicine, Department of Radiation Oncology, University Hospital Bonn, Rheinische Friedrich-Wilhelms-Universität Bonn, 53127 Bonn, Germany

**Keywords:** Predictive preventive personalized medicine (3PM/PPPM), Phytochemicals, Flavonoids, Flavanones, Flavonols, Flavones, Flavanols, Isoflavonoids, Chalcones, Anthocyanidins, Anti-cancer agents, Drug-sensitizing effect, Targeted therapy, Radiotherapy, Chemotherapy, Immunotherapy, Therapy resistance, Anti-inflammation, Anti-bacterial, Anti-viral, COVID-19, Signalling pathways, Therapy efficacy, Nano-carrier delivery, Disease management, Health economy, Health policy

## Abstract

Cost-efficacy of currently applied treatments is an issue in overall cancer management challenging healthcare and causing tremendous economic burden to societies around the world. Consequently, complex treatment models presenting concepts of predictive diagnostics followed by targeted prevention and treatments tailored to the personal patient profiles earn global appreciation as benefiting the patient, healthcare economy, and the society at large. In this context, application of flavonoids as a spectrum of compounds and their nano-technologically created derivatives is extensively under consideration, due to their multi-faceted anti-cancer effects applicable to the overall cost-effective cancer management, primary, secondary, and even tertiary prevention. This article analyzes most recently updated data focused on the potent capacity of flavonoids to promote anti-cancer therapeutic effects and interprets all the collected research achievements in the frame-work of predictive, preventive, and personalized (3P) medicine. Main pillars considered are:

- Predictable anti-neoplastic, immune-modulating, drug-sensitizing effects;

- Targeted molecular pathways to improve therapeutic outcomes by increasing sensitivity of cancer cells and reversing their resistance towards currently applied therapeutic modalities.

## Introduction

### Flavonoids in primary, secondary, and tertiary anti-cancer management

By evidence, a large portion of malignancies is considered as being preventable, and a cost-effective targeted anti-cancer protection is an issue in the overall management of the disease [1]. To this end, an application of natural substances is an attractive strategy for primary and secondary cancer prevention [2]. Preclinical cancer research demonstrates potent genoprotective properties of flavonoids in non-cancer models. Corresponding mechanisms applicable to the primary and secondary anti-cancer care include multi-faceted effects such as anti-Warburg cell transformation and anti-mitochondriopathic activities, anti-inflammatory, antibacterial, and anti-viral, among other health-beneficiary effects [3–5].

In tertiary care, flavonoids have been proposed to protect patients against poor outcomes in case of COVID-19 infection, due to their evident anti-inflammatory, anti-bacterial, and anti-viral properties [6, 7].

Protective anti-cancer application of flavonoids in the context of 3P medicine should follow principles of the evidence-based therapeutic effects, individualized prediction, targeted prevention, and personalization of the treatment algorithms. To this end, application of specialized analytical approaches towards a companion diagnostics is strongly recommended such as liquid biopsy analysis, risk assessment tools, multi-omics and multi-parametric analysis, and application of artificial intelligence in medicine [8].

### Radiotherapy and chemotherapy as hallmarks of anti-cancer treatments: status quo

Radiotherapy and chemotherapy are hallmarks of the currently applied anti-cancer treatments [9]. Conventional anti-cancer strategies pose several deficits [10, 11]. Despite recent progress in anti-cancer strategies, the development of resistance remains the leading cause of cancer-related mortality, and many patients develop resistance towards anti-cancer agents applied [12]. Moreover, radiotherapy, chemotherapy, and targeted therapy require effective blood flow into the tumor microenvironment; perfusion deficits reduce overall therapeutic efficacy [13]. An improved understanding of carcinogenic processes allows for the technological innovation creating more efficient therapeutic modalities [10]. Targeted anti-cancer therapies are expected to leverage unique molecular changes associated with specific cancer types [14, 15].

Cancer resistance can be classified into primary and acquired. Primary (or intrinsic) cancer resistance exists before the commencement of treatment [16]. It can be caused by (a) pre-existing genetic mutations in tumors resulting in decreased responsiveness to therapy, such as in the case of triple-negative breast cancer (TNBC); (b) heterogeneity of tumors with pre-existing insensitive subpopulations such as cancer stem cells (CSCs); or (c) activation of intrinsic pathways as defenses against chemotherapeutic drugs [17]. In contrast, acquired resistance develops after the initial therapy [16] and is characterized by a gradual reduction in anti-cancer efficacy. Acquired drug resistance can result from the activation of newly emerged driver genes, mutations/altered expression of drug targets, or changes in the tumor microenvironment after treatment [17]. Current statistics of the World Health Organization (WHO) indicate that one in every six deaths worldwide is due to cancer. Therefore, in a view of the consequent severe socio-economic burden to the society, it is necessary to shift the paradigm of cancer management from reactive to predictive, preventive, and personalized medical approaches [18, 19]. Contextually, the focus of current cancer research on the identification of molecules could improve individual outcomes of cancer treatments, including overcoming resistance and increasing the sensitivity of cancer cells towards treatments applied.

Phytochemicals, isolated or in corresponding intact plants, are a verified source of natural anti-cancer molecules targeting a variety of pathways associated with neoplastic transformation and cancer progression [2, 20–41]. Indeed, flavonoids exert significant anti-cancer effects in clinical and preclinical studies [3, 42–47]. Therefore, we emphasize the great evidence-based potential of bioactive flavonoids in modulating cancer cells’ response to anti-cancer drugs by overcoming resistance and/or sensitizing cancer cells to currently applied therapies.

### Focus of the current study: Flavonoids as a helper in anti-cancer therapy

This review focuses on flavonoids’ capacity to modulate the responsiveness of cancer to conventional treatment modalities. Of particular interest are the helper effects against cancer therapy resistance by sensitizing malignant cells towards therapies. Those properties of flavonoids clearly demonstrated in preclinical studies are considered of particularly great clinical utility, when applied to anti-cancer therapies tailored to the personalized patient profile [48–59].

### Source of the analyzed research data

Data were collected from the biomedical literature sources utilizing “resistance” and “flavonoids” or “flavanones” or “flavonols “ or “flavones” or “flavanols” or “isoflavonoids” or “chalcones” or “anthocyanidins” and “radiotherapy” or “chemotherapy” or “targeted therapy” or other relevant items as either keywords or medical subject heading (MeSH) terms in searches of the PubMed database. Most recently updated research published within the time-frame of 2018–2021 years has been taken into consideration for the below presented data analysis and interpretation.

## Anti-cancer therapy: from conventional to advanced approaches

The development of radiotherapy to treat cancer in the early 1900s was followed by the discovery of the chemotherapy. The breakthrough of modern oncology was introduced by targeted therapies directed at specific tumor and molecular alterations and immune checkpoint inhibitors to stimulate the immune system against cancer [9]. Therefore, oncology research in the last 20 years has yielded new anti-tumor strategies—including monoclonal antibodies and immunotherapeutic agents—that significantly increase treatment efficacy and allow personalized, highly effective, and less toxic approaches for individual patients [9]. Unfortunately, the initial favourable response to treatment is often followed by the development of resistance that reduces therapeutic efficacy and leads to cancer relapse and recurrence [60]. However, further progress in precision medicine in the twenty-first century enables an individualized approach in the context of predictive, preventive, and personalized medicine that can improve cancer management [18, 61–66]. Cancer treatment can also be improved by applying natural substances, which sensitize cancer cells to therapeutic agents [67–70].

## Flavonoids: origin and classification

**Fig. 1:**
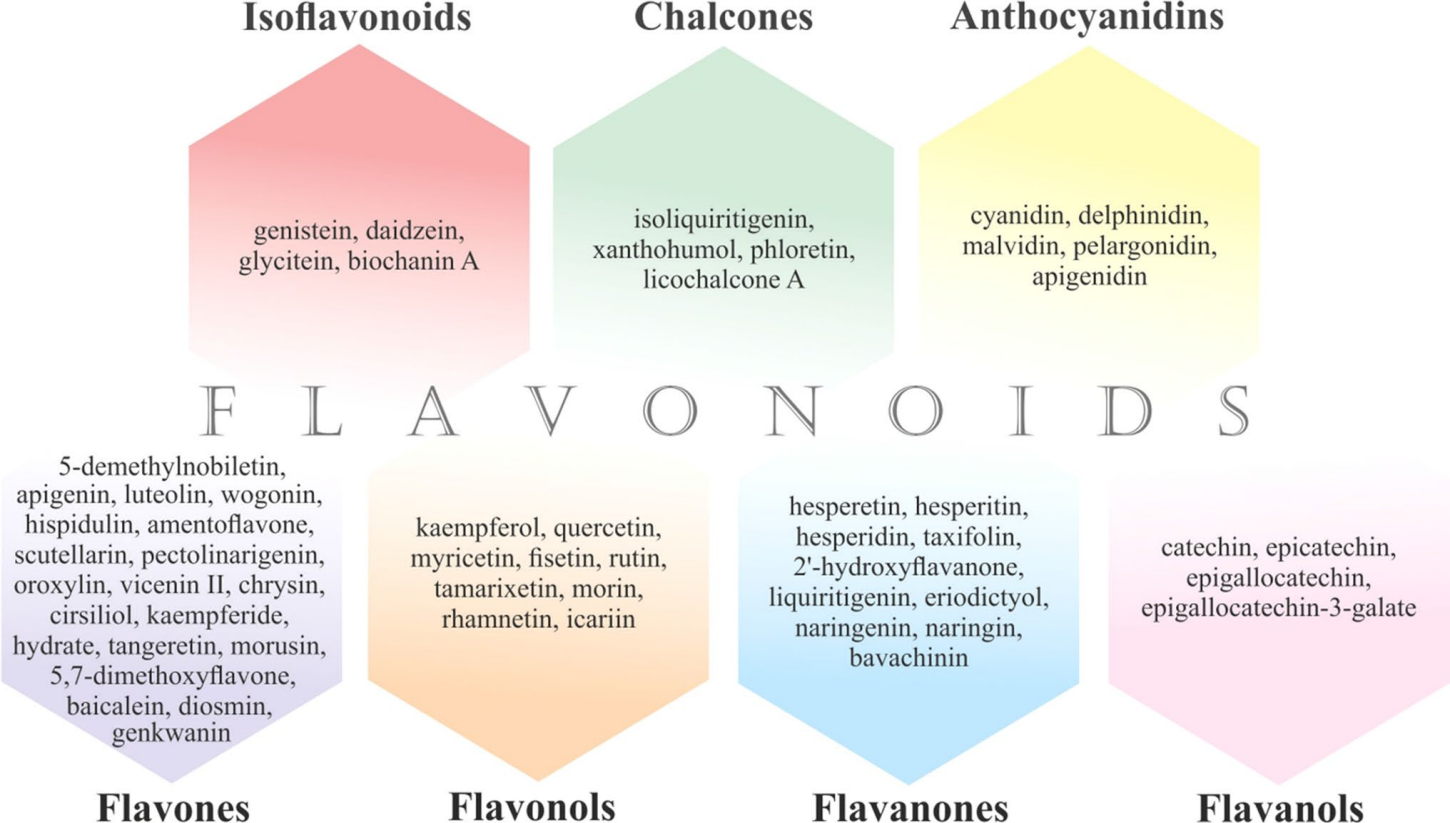
Classification of flavonoids

lavonoids are phenolic compounds widely found in vegetables, fruits, beverages, nuts, olive oil, red wine, and medicinal plants [3, 7, 46, 47, 71, 72]. Chemically, flavonoids have fifteen-carbon skeletons consisting of two benzene rings connected through a pyrane ring. The classification of flavonoids is based on the chemical structure, oxidation level, and substitution pattern of the heterocyclic pyrane ring (C ring) [7]. The classification of flavonoids is provided in Fig. 1 [3, 7, 71, 73–79].

Flavonoids exert numerous biological effects, including antioxidant, anti-inflammatory, anti-mutagenic, and anti-cancer activities, among others [46]. Although flavonoids have demonstrated significant anti-cancer efficacy in preclinical research [46, 69, 71, 75, 76], clinical studies evaluating the anti-cancer effects of flavonoids remain sparse. Genistein was demonstrated to be safe and tolerable in combination with chemotherapy in a recent phase I/II pilot study [43]. Other clinical trials revealed the potential of flavonoids as anticarcinogenic agents [80] and as complementary antitumor agents in colorectal cancer patients [45]. However, the significant capacity of flavonoids to improve therapeutic outcomes by improving the treatment sensitivity or reversing the resistance of cancer cells to anti-cancer therapeutic agents is currently evaluated predominantly in preclinical in vitro and in vivo research [67–70].

## Radiotherapy resistance

**Fig. 2:**
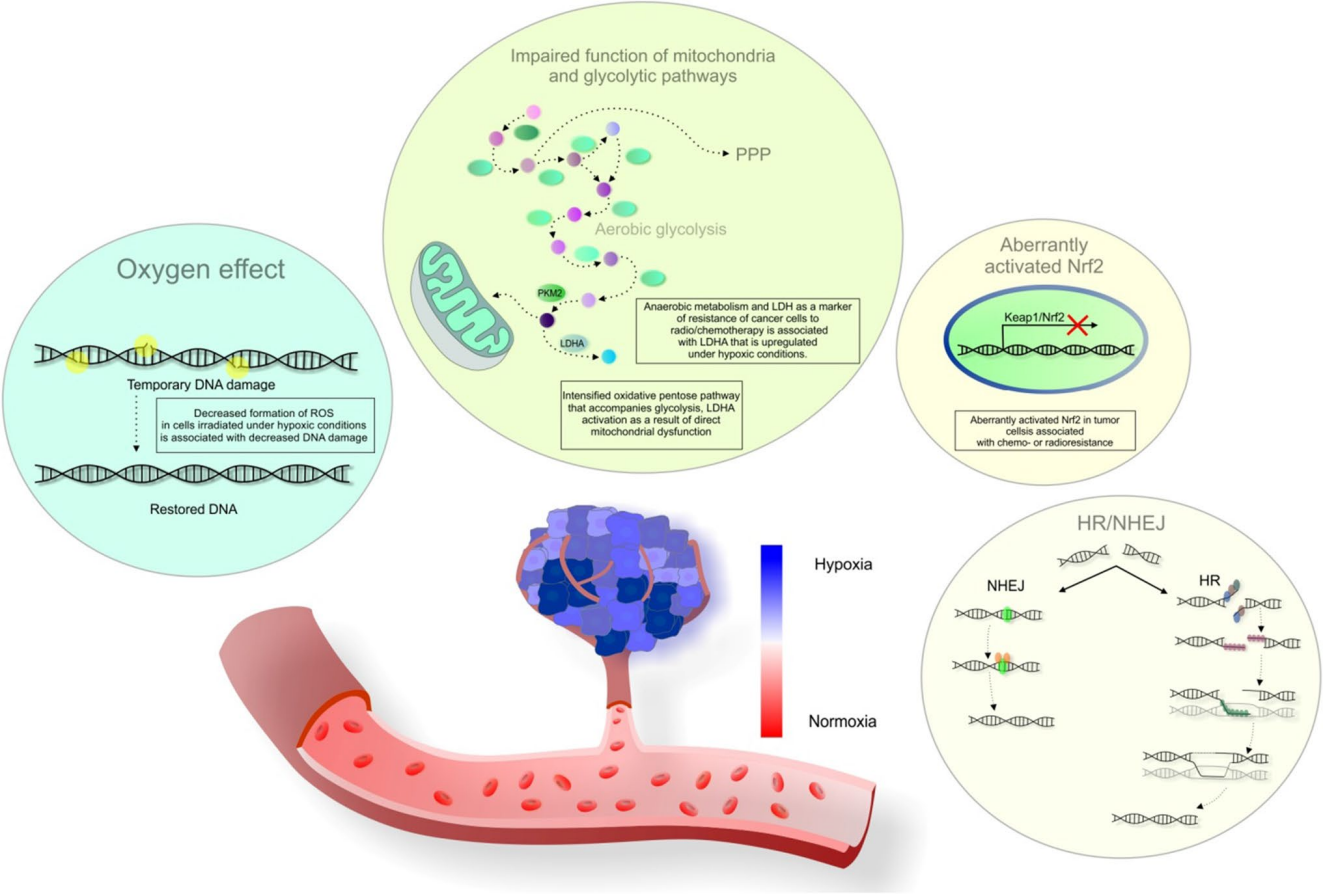
Mechanisms of radiotherapy resistance of cancer cells. *Explanatory notes*: A hypoxic intratumoral microenvironment is a leading cause of radiotherapy failure (decreased ROS formation in irradiated cells under hypoxic conditions is associated with decreased DNA damage and the so-called “oxygen effect”). Indeed, impaired function of mitochondria and glycolytic pathways can be involved in cancer cell radioresistance (anaerobic metabolism and LDH, a marker of resistance, associated with upregulated LDHA under hypoxic conditions). LDH is a marker of perfusion-related hypoxia. Lower oxygen leads to reductions in radiation-induced ROS generation and DNA damage. Upregulation of the oxidative pentose pathway that accompanies glycolysis, activation of LDHA as a result of direct mitochondrial dysfunction or oncogene/HIF-mediated inactivation of mitochondrial function, and inhibition of pyruvate entry into mitochondria by pyruvate-dehydrogenase kinases (regulated by LDHA through HIF) are processes associated with cancer cells radioresistance [13]. Also, aberrantly activated Nrf2 in tumor cells (as a result of *Keap1* or *Nrf2* somatic mutations or other Keap1/Nrf2-related mechanisms) contributes to high-level resistance of cancer cells [82]. The *Keap1* promoter is often hypermethylated in NSCLS and leads to decreased Keap1 mRNA and protein expression; this impairs the Nrf2-Keap1 pathway (resulting in radio- and/or chemo-resistance) [83]. The homologous recombination (HRR) and non-homologous end joining (NHEJ) pathways enhance DNA repair activity and modulate cell sensitivity and resistance to radiotherapy [48]. The repair of DNA damage in dormant cancer stem cells (CSCs) is predominantly performed through NHEJ; consequently, NHEJ inhibition could overcome CSC radioresistance [84]. Indeed, CSCs are considered the primary source of resistance to radiotherapy and chemotherapy while tumor heterogeneity contributes to radiation resistance [11].

Radiotherapy is a component of multidisciplinary treatment regimens applicable to various cancer types [81]. Up-to-date technologies enable the precise delivery of radiation to tumor lesions with minimal injury to healthy tissue. However, many cancer types are associated with insensitivity to radiotherapy due to intrinsic resistance or recurrence after treatment due to acquired resistance [11]. Figure 2 provides a detailed overview of specific mechanisms related to cancer cells’ radiotherapy resistance, including a predominantly hypoxic tumor microenvironment and the consequent modulation of mitochondrial and glycolytic pathways, Keap1/Nrf2-related mechanisms, homologous recombination (HRR), and non-homologous end joining (NHEJ).

### Flavonoids sensitize cancer cells to radiotherapy

While flavonoids have radioprotective effects on healthy cells, they are considered potent radiosensitizing molecules of cancer cells [85]. Genistein mediated selective radiosensitizing effects in non-small cell lung cancer (NSCLC) A549 cells by inhibiting the methylation of the *Keap1* gene promoter region; hypermethylation of the *Keap1* promoter results in chemo/radioresistance mediated by the Nrf2-Keap1 pathway [83]. Also, genistein enhanced the radiosensitivity of NSCLC A549 cells, as demonstrated through increased apoptosis and Beclin-1-induced autophagy by inhibiting Bcl-xL and Bcl-xL-Beclin-1 interactions [86]. Moreover, apigenin and the terpenoid cryptotanshinone exerted synergistic radiosensitizing effects in the in vivo murine model of Ehrlich carcinoma, as demonstrated by the downregulation of angiogenic and lymphangiogenic regulators and the induction of apoptosis [87]. Furthermore, genistein and the tyrosine kinase inhibitor AG1024 (tyrphostin) synergistically increased the radiosensitivity of prostate cancer PC3 and DU145 cells by suppressing the homologous recombination (HRR) and non-homologous end joining (NHEJ) pathways [48]. Breast safeguard (BSG) is a commercial nutrient supplement composed of several phytochemicals, including but not limited to flavonoids (genistein, quercetin, indol-3-carbinol, resveratrol, C-phycocyanin, gallic acid, and curcumin). BSG attenuated the responsiveness of hepatocellular carcinoma (HCC) HepG2 cells to ionizing radiation leading to the inhibition of proliferation, survival, and migration [88]. Also, quercetin pre-treatment enhanced colon cancer HT-29 and DLD-1 cells’ radiosensitivity through Notch-1 and CSC targeting [89]. Moreover, Koh et al. recently evaluated the effects of baicalein on TNBC MDA-MB-231/IR cells obtained by irradiating the parental MDA-MB-231 cells with 2 Gy irradiation; MDA-MB-231 cells are characterized by enhanced migration, invasion, and stem-cell-like properties. Indeed, baicalein reduced chemo- and radio-resistance, induced apoptosis, and suppressed stem cell-like properties in MDA-MB-231/IR cells; baicalein also reversed the expression of interferon-induced protein with tetratricopeptide repeats 2 (IFIT2), which is involved in cancer metastasis and recurrence. However, further studies are needed to evaluate the mechanisms of IFIT2 in resistant breast cancer cells [49].

Table 1 provides a detailed overview of the specific mechanisms through which flavonoids enhance radiotherapy.

**Table 1:** Flavonoids enhance the efficacy of radiotherapy: Up-to-date evidence

Flavonoids	Study details	Effects	Mechanisms	Reference
Genistein	NSCLC A549 cells	Radiosensitizing effects	Inhibition of the methylation of the *Keap1* gene promoter region	[83]
Apigenin and terpenoid cryptotanshinone	Apigenin and/or cryptotanshinone intraperitoneally injected into non-irradiated or γ-irradiated solid Ehrlich carcinoma-bearing mice	Radiosensitizing effects	Downregulation of angiogenic and lymphangiogenic regulators (signal transducer and activator of transcription 3, VEGF-C, and TNF-α, suppressing MMP-2 and -9 activities) and induction of apoptosis (enhancing apoptosis by inducing cleaved caspase-3 and granzyme B levels)	[87]
Genistein	NSCLC A549 cells	Enhanced radiosensitivity	Increased apoptosis and Beclin-1-induced autophagy (inhibition of Bcl-xL and Bcl-xL-Beclin-1 interactions)	[86]
Genistein and tyrosine kinase inhibitor (tyrphostin) AG1024	Prostate cancer PC3 and DU145 cells	Enhanced radiosensitivity	Suppressed HRR and NHEJ pathways	[48]
BSG	HCC HepG2 cancer cells	Attenuated responsiveness to ionizing radiation leading to the inhibition of proliferation, survival, and migration		[88]
Quercetin	Colon cancer HT-29 and DLD-1 cells	Enhanced radiosensitivity	Notch-1 and CSC targeting	[89]
Baicalein	TNBC MDA-MB-231/IR cells	Sensitization of chemo- and radio-resistant cells	Induced apoptosis and suppressed stem cell-like properties (mammosphere formation, side population, expression of Oct3/4 and ABCG2, and CD44^high^CD24^low^ population); reversed IFIT2 expression	[49]

*ABCG2* breast cancer resistance protein (BCRP); *BSG* Breast safeguard; *CSC* cancer stem cells; *HRR* homologous recombination; *IFIT2* interferon-induced protein with tetratricopeptide repeats 2; *MDA-MB-231/IR cells* triple-negative breast cancer cells obtained by irradiating the parental MDA-MB-231 cells with 2 Gy irradiation; *NHEJ* non-homologous end joining; *TNBC* triple-negative breast cancer; *TNF-α* tumor necrosis factor α; *VEGF-C* vascular endothelial growth factor-C; *MMP-2/-9* matrix metalloproteinase-2/-9

## Anti-cancer chemotherapy resistance

After the accidental discovery of the first DNA alkylating agent in the 1940s, several chemotherapeutic modalities were developed, becoming the first revolutionary anti-cancer pharmacological approach [9]. These include alkylating agents (the triazene compounds dacarbazine and temozolomide and the metal salts cisplatin, carboplatin, and oxaliplatin), antimetabolites (the folate analogs aminopterin and methotrexate, the purine analog mercaptopurine, and the pyrimidine analogs 5-fluorouracil, gemcitabine, and capecitabine), antimitotics (vincristine, the topoisomerase I inhibitors topotecan and irinotecan, and the microtubule-stabilizing molecules paclitaxel, docetaxel, and cabazitaxel), cytotoxic antibiotics and related substances (daunomycin/daunorubicin, actinomycin D, and doxorubicin), polyamine inhibitors and iron-modulating drugs (ciclopirox and triapine), and combination chemotherapy regimens [9]. Chemotherapeutic agents target cancer cells and all rapidly dividing cells [14] and are often associated with primary or acquired resistance [9].

**Fig. 3:**
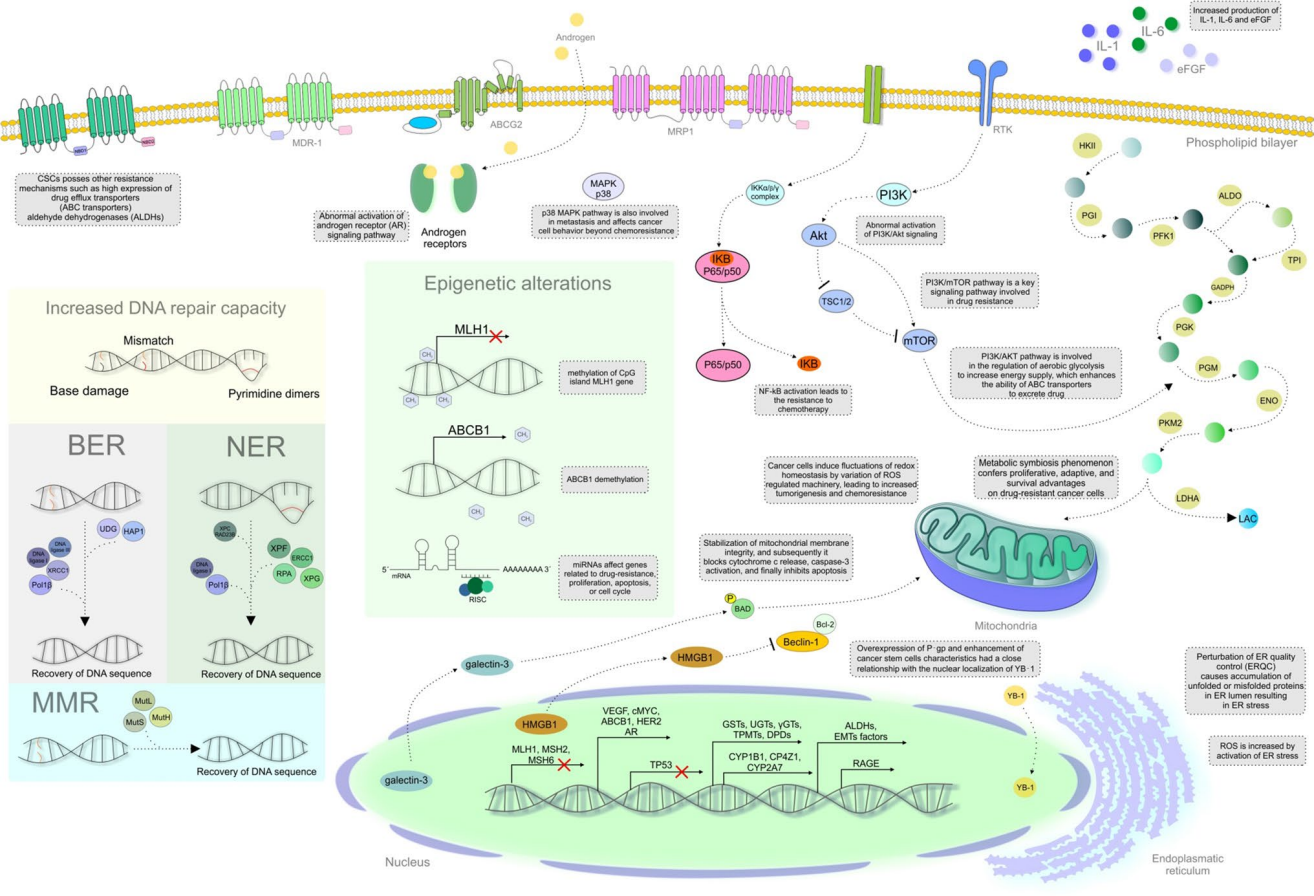
Mechanisms of resistance to chemotherapeutic drugs. *Explanatory notes*: **A) increased drug efflux** – proteins in the ATP-binding cassette (ABC) transporter family contain nucleotide-binding domains (NBD) and two transmembrane domains (TMDs); ATP hydrolysis-driven conformational changes of TMD result in unidirectional transport across the lipid bilayer [91]. ABC transporter overexpression is observed in several cancer types and is more predominant in cancer stem cells (CSCs) [92]. ABC transporters, including multidrug resistance protein 1 (MDR-1, ABCB1, P-gp), multidrug resistance-associated protein 1 (MRP1, ABCC1), and breast cancer resistance protein (BCRP, ABCG2), are implicated in drug-resistant cancers [93]. Also, aldehyde dehydrogenase (ALDH) promotes drug resistance. ABC transporters and ALDH are upregulated in normal stem cells, CSCs, and drug-resistant cancer cells [94]. ***B) Increased DNA repair capacity*** [95] ***or tolerance of DNA damage*** [96] induced by therapeutic agents [95, 96]—base excision repair (BER) involves different proteins (UDG, HAP1, Polβ, XRCC1, and DNA ligase I or III) [96] and nucleotide excision repair (NER) mechanisms involve damage recognition/excision proteins and helicase proteins (*DNA damage is recognized by the NER protein XPC-RAD23B, which binds to DNA strand, an oligonucleotide containing the lesion is then excised from the DNA strand, a repair patch is synthesized, and DNA ligases join the patch to the DNA*) [96]. NER-induced resistance to platinum-based agents [12, 96] includes the DNA repair endonuclease XPF and the DNA excision repair protein ERCC1. Replication protein A (RPA) is involved in the DNA-damage response (DDR), HR, and NER [12]. Decreased mismatch repair (MMR) promotes damage tolerance and enhanced mutagenicity and chemoresistance in cancer cells (*hypermethylation of the hMLH1 gene promoter results in decreased expression of the MLH1 protein involved in the MMR pathway*) [12]. ***C) Genetic and epigenetic factors***—TP53 loss results in continued replication and resistance to genotoxic drugs [12]. Abnormal activation of the androgen receptor (AR) signaling pathway (AR over-expression, AR gene amplification, mutations, alterations in coregulators, and continuous androgen release from the tumor tissue or adrenal glands) and abnormal activation of PI3K/Akt or PI3K/Akt/mTOR signaling can lead to the overexpression of ABC transporters and the upregulation of oncogenes and growth factors such as VEGF and c-myc. The acidified tumor micro-environment promotes aerobic glycolysis and MDR (by reducing drug absorption and efficiency). PI3K/Akt regulates aerobic glycolysis to increase energy supply and enhance ABC transporter-mediated drug excretion [97]. The transcription of specific genes essential for resistance is enhanced (e.g. *ABCB* amplification) [12, 98, 99]. Epigenetic alterations (genome-wide DNA hypomethylation, regional hypermethylation, changes in histone modifications, and alterations in miRNA expression) [12] – carboplatin-induced methylation of the *MLH1* CpG island (important for the MMR DNA repair system) is associated with chemoresistance in ovarian cancer; *ABCB1* demethylation decreases the accumulation of anti-cancer drugs and promotes the acquisition of the multidrug phenotype [12]. ***D) Growth factors***—cytokine (IL-1, IL-6) production is increased in multidrug cancer cells when compared with drug-sensitive cancer cells [12]. Specific chemotherapeutic agents were ineffective against cancers with increased levels of extracellular fibroblast growth factors (eFGF) [12]. ***E) Increased metabolism of xenobiotics***—altered expression of isoforms of cytochrome (CYPs)—overexpressed CYP1B1, CP4Z1, CYP1B1, and CYP2A7 and phase II enzymes, such as glutathione-S-transferases (GSTs), uridine diphospho-glucuronosyltransferases (UGTs), gamma-glutamyl transferases (γGTs), thiopurine methyltransferases (TPMTs), and dihydropyrimidine dehydrogenases (DPDs) promote the development of multidrug resistance (MDR) [12]. ***F) CSCs***—targeted less by chemotherapeutic drugs (due to slow cell cycle kinetics, high expression of ABC transporters, ALDHs, epithelial-mesenchymal transition, and factors affecting the tumor microenvironment, such as hypoxia, and epigenetic modifications) [100]. ***F) Other mechanisms include endoplasmatic reticulum (ER) stress***—perturbation of ER quality control (ERQC) causes the accumulation of unfolded or misfolded proteins in the ER lumen, resulting in ER stress. The ER stress response (ERSR) is produced to restore homeostasis or activate cell death. ERS is critical for chemo-therapeutic resistance, following the initiation of an ERSR [101]. ROS is increased by the activation of ER stress. Cancer cells induce fluctuations of redox homeostasis through the variation of ROS-regulated machinery, leading to increased tumorigenesis and chemoresistance [102]. ***The receptor for advanced glycation end products (RAGE)*** activation leads to drug resistance (pancreatic cancer) [103]. P‐gp overexpression and CSCs are closely associated with the nuclear localization of ***YB‐1*** in cancer cells [50]. ***NF-kB activation*** rescues cancer cells from cell death [104]. ***Galectin-3*** is transported from the nucleus to the cytoplasm to stimulate the phosphorylation of Bcl-2 associated death (Bad) protein and the downregulation of Bad; this results in the maintenance of mitochondrial membrane integrity. Consequent effects, including the blockade of cytochrome c release and caspase-3 activation, inhibition of apoptosis, and activation of ***PARP1***, induce chemoresistance through the cytosolic translocation of HMGB1 via PARylation, which is known to induce autophagy by disrupting the interaction between Beclin-1 and Bcl-2 [51]

Cancer cells gradually develop resistance to almost all chemotherapeutics through various mechanisms. Cancer drug resistance is associated with increased drug efflux, alterations in drug metabolism, transport, and signal transduction molecules, elevated DNA repair capacity and apoptotic evasion, increased mutations, reactivation of drug targets, crosstalk with the cancer microenvironment and cancer cell-stroma interactions, epithelial-mesenchymal transition (EMT)-mediated chemoresistance, epigenetic mechanisms, metabolic alteration, and the effects of CSCs [16, 90]. Despite initial responses to therapy due to the majority of cells being sensitive to the drug, the pre-existence of resistant cell subpopulations can result in relapse after chemotherapy. Resistant CSCs are involved in chemotherapy resistance in various cancer types. Intrinsic resistance can be mistaken with acquired, as resistance seems to be acquired due to therapy [17]. Both resistance factors interact and jointly modulate drug resistance. Indeed, 90% of cancer progression during and after chemotherapy is associated with drug resistance [90]. Prolonged administration of a chemotherapeutic agent can result in resistance to multiple other structurally unrelated agents, a phenomenon known as multidrug resistance (MDR) [16]. Therefore, it is necessary to provide new strategies to overcome cancer cells’ resistance to chemotherapeutic agents [90]. Figure 3 provides a detailed overview of specific mechanisms related to cancer cell drug resistance. One key mechanism involves increased drug efflux associated with the overexpression of aldehyde dehydrogenase (ALDH) and the ATP-binding cassette (ABC) transporter family of proteins. This is associated with drug resistance, increased DNA repair capacity and tolerance to DNA damage, genetic factors such as abnormal activation of the androgen receptor (AR) signalling pathway, PI3K/Akt signalling, epigenetic factors, increased xenobiotic metabolism, and other mechanisms including contributions from endoplasmatic reticulum (ER) stress, the receptor for advanced glycation end products (RAGE), NF-kB, and galectin-3.

Current research highlights the potential of co-administration of natural compounds such as flavonoids with chemotherapeutic agents as an attractive strategy to overcome chemotherapeutic resistance and MDR in tumors [105].

### Flavonoids enhance effectiveness of conventional chemotherapeutic agents

The resistance or insensitivity of cancer cells to chemotherapeutics is a serious disadvantage of cytotoxic anti-cancer therapies. However, current research highlights the potential importance of flavonoids in increasing the sensitivity and/or efficacy of chemotherapeutic agents.

Several flavonols, including quercetin, kaempferol, or morin, exert potent capacities to modulate cancer cell chemoresistance [50–53, 98, 103, 106, 107]. Abnormal activation of AR and PI3K/Akt signaling is considered a significant cause of docetaxel resistance. Interestingly, quercetin revealed docetaxel resistance-reversing effects in docetaxel-resistant prostate cancer (LNCaP/R, PC-3/R) cells in vitro and in a prostate cancer xenograft model in vivo by reversing the upregulation of P-gp, the development of mesenchymal and stem-like cell phenotypes, and the activation of androgen receptor and PI3K/Akt signaling pathways; moreover, the combinatory treatment of quercetin and docetaxel slowed tumor growth and robustly inhibited proliferation in vivo [98]. Similarly, quercetin enhanced the therapeutic efficiency of paclitaxel in prostate cancer PC-3 cells in vitro through the induction of ER stress and ROS production; this combinatory treatment also exerted beneficial effects in a PC-3 cancer-bearing murine model in vivo [107]. Also, quercetin promoted cell death and gemcitabine sensitivity in human pancreatic cancer MIA Paca-2 and MIA Paca-2 (GEM-resistant) cells through the receptor for advanced glycation end products (RAGE)/PI3K/AKT/mTOR axis, especially through RAGE inhibition [103]. Quercetin also enhanced doxorubicin, paclitaxel, and vincristine activity and thus reversed MDR in breast cancer MCF-7 and doxorubicin-resistant MCF-7 (MCF-7/Dox) cells by downregulating P-gp and eliminating CSCs through YB-1 nuclear translocation [50].

Kaempferol combined with 5-FU exerted a synergistic inhibitory effect on cell viability, enhanced apoptosis, and induced cell cycle arrest in both chemo-resistant and sensitive colon cancer LS174 cells. Kaempferol also blocked the production of reactive oxygen species (ROS) and modulated the expression of JAK/STAT3, MAPK, PI3K/AKT, and NF-κB signaling in these cells [52]. As discussed above, MDR, a state of certain cancers becoming cross-resistant to structurally diverse antineoplastic agents, is associated with the overexpression of ABC transporters [108]. However, kaempferol exerted an ability to inhibit MDR by downregulating ABCB1, ABCC1, Akt, and BCL2 in leukemia HL-60 and NB4 cells [53].

Co-treatment with morin and cisplatin led to the synergistic sensitization of ovarian cancer SK-OV-3 (cisplatin-resistant) cells to cisplatin. Further, the sensitization of ovarian cancer cells to cisplatin is suggested to be achieved through the downregulation of galectin-3 (essential for various cellular processes such as apoptosis) by morin [106]. Moreover, morin hydrate reversed the acquired resistance of cisplatin-resistant hepatocellular cancer HepG2^DR^ cells by impairing PARP-1/HMGB1-dependent autophagy; indeed, PARP-1 autophagy appears to be regulated by the PARylation of HMGB1 [51].

In addition to flavonols, the flavanone hesperetin sensitized cisplatin (DDP)-resistant human lung cancer cells (A549/DDP) to cisplatin in vivo and in vitro, mechanistically through decreased expression of P‑gp and increased intracellular accumulation of the P‑gp substrate, rhodamine 123 [109]. Similarly, poncirin, a flavanone glycoside with a bitter taste, enhanced sensitivity to cisplatin by decreasing the expression of MDR-1, MRP1, and BCRP and inhibiting PI3K/Akt signaling in cisplatin-resistant osteosarcoma (OS) cells [110].

Moreover, other classes of flavonoids such as chalcones also exhibit potent chemosensitizing capacities in cancer models. The combination of xanthohumol, a prenylated flavonoid from hops, and the chemotherapeutic agent SN38, the active metabolite of irinotecan, in resistant colon cancer SW480 cells decreased cell viability compared with SN38 alone. Therefore, xanthohumol can be potentially utilized as a chemosensitizer of SN38 [111]. Another chalcone, flavokawain-B, showed potent anti-cancer abilities in gemcitabine-resistant NSCLC cells by inducing apoptosis and ROS production and blocking the PI3K/Akt signalling pathway [112].

Furthermore, Fan et al. recently evaluated the inhibitory effects of flavonoids on breast cancer resistance protein (BCRP) in vitro and in vivo. Eleven flavonoids (amentoflavone, apigenin, biochanin A, chrysin, diosmin, genkwanin, hypericin, kaempferol, kaempferide, licochalcone A, and naringenin) significantly inhibited BCRP in BCRP-overexpressing (BCRP-MDCKII) cells. Simultaneously, these effects were associated with reduced BCRP-mediated doxorubicin and temozolomide efflux and increases in the drugs’ cytotoxicity. Also, mitoxantrone’s co-administration with the above-mentioned flavonoids promoted the AUC_0-t_ of mitoxantrone to different extents in a Sprague–Dawley rat model [113].

Moreover, dihydromyricetin, a natural flavonoid from the leaves of *Vitis heyneana*, reversed MRP2-induced MDR by preventing NF-κB-Nrf2 signaling in colorectal cancer HCT116/OXA and HCT8/VCR cell lines [114]. Also, bavachinin, tephrosin, and candidone sensitized MDR MCF7/MX and EPG85.257RDB cells to daunorubicin and mitoxantrone [115].

Table 2 provides a detailed overview of specific mechanisms through which flavonoids enhance the therapeutic efficacy of conventional chemotherapeutic agents. These results suggest a significant potential of increased therapeutic efficacy through a combination of flavonoids and conventional chemotherapeutic agents.

**Table 2:** Flavonoids enhancing the efficacy of conventional chemotherapeutic agents: Up-to-date evidence

Flavonoid	Study details	Effect	Mechanisms	Reference
Quercetin 20 μM (+ Docetaxel 5 nM)	Prostate cancer xenograft models (LNCaP/R and PC-3/R cells injected into nude mice) and docetaxel-resistant prostate cancer cells (LNCaP/R, PC-3/R)	Docetaxel-resistance reversal	Reversed the upregulation of P-gp, the development of mesenchymal and stem-like cell phenotypes, and the activation of androgen receptor and PI3K/Akt signalling Slower growth of tumors and strongest proliferation inhibitory effects (Ki67)	[98]
Quercetin (+ Paclitaxel)	Prostate cancer cells (PC-3) and PC-3 cancer-bearing murine model	Enhanced therapeutic efficiency of paclitaxel	Induced ER stress and ROS generation (also inhibited cell proliferation and migration, increased apoptosis, and arrested the cell cycle at the G2/M)	[107]
Quercetin (+ Gemcitabine)	Pancreatic cancer MIA Paca-2 and MIA Paca-2 (GEM-resistant) cells	Promoted sensitivity to gemcitabine and increased cell death	Modulation of the RAGE/PI3K/AKT/mTOR axis, especially via RAGE inhibition	[103]
Quercetin (+ Doxorubicin/Paclitaxel/Vincristine)	Breast cancer MCF-7 and doxorubicin resistant MCF-7 (MCF-7/dox) cells	Enhanced the activity of doxorubicin, paclitaxel, and vincristine (reversed MDR)	P-gp downregulation and elimination of cancer stem cells through YB-1 nuclear translocation	[50]
Kaempferol (+ 5-FU)	5-FU resistant LS174 colon cancer cells	Sensitized cancer cells to 5-FU	Modulated JAK/STAT3, MAPK, PI3K/Akt, and NF-κB signalling blockaded ROS production, inhibited cell viability, and enhanced apoptosis and cell cycle arrest	[52]
Kaempferol	Leukemia HL-60 and NB4 cells	Inhibited MDR (and promoted apoptosis)	Decreased ABCB1, ABCC1, Akt, and BCL2	[53]
Morin (+ Cisplatin)	Ovarian cancer SK-OV-3 (cisplatin-resistant) cells	Increased sensitization to cisplatin	Reduced galectin-3	[106]
Morin hydrate (+ Cisplatin)	Hepatocellular cancer cells HepG2^DR^	Reversed cisplatin resistance	Impaired PARP1/HMGB1-dependent autophagy	[51]
Hesperetin (+ Cisplatin)	Cisplatin (DDP)-resistant human lung cancer cells (A549/DDP) in vitro and in vivo (A549/DDP cell injected into nude mice)	Increased sensitization to cisplatin	Decreased P‑gp, increased intracellular accumulation of rhodamine 123, and inhibited NF-κB activation	[109]
Poncirin (+ Cisplatin)	Cisplatin resistant osteosarcoma cells	Enhanced sensitivity to cisplatin	Decreased MDR-1, MRP1, and BCRP, and inhibited PI3K/Akt signalling	[110]
Xanthohumol (+ SN38)	Resistant colon cancer SW480 cells	Chemosensitization to SN38	Decreased cancer cell viability	[111]
Flavokawain-B	Gemcitabine-resistant NSCLC cells	Targeting of gemcitabine-resistant cancer cells	Induced apoptosis and ROS production, and blocked the PI3K/Akt signalling pathway	[112]
Eleven flavonoids: amentoflavone, apigenin, biochanin A, chrysin, diosimin, genkwanin, hypericin, kaempferol, kaempferide, licochalcone A, naringenin (+ doxorubicin/temozolomide/mitoxantrone)	BCRP-MDCKII cells and Sprague–Dawley rat model	Reduced efflux of doxorubicin and temozolomide	Reduced BCRP-mediated efflux of doxorubicin and temozolomide and increased cytotoxicity; Promoted AUC_0-t_ of mitoxantrone to different extents in vivo	[113]
Dihydromyricetin	Colorectal cancer HCT116/OXA and HCT8/VCR cell lines	Reversed MRP-2-induced MDR	Prevented NF-κB-Nrf2 signalling	[114]
Bavachinin, tephrosin, and candidone	MDR MCF7/MX and EPG85.257RDB cells	Sensitization of MDR cells daunorubicin and mitoxantrone		[115]

*5-FU* 5-Fluorouracil; *BCRP* breast cancer resistance protein (ABCG2); *BCRP-MDCKII* BCRP-overexpressing; *MDR* multidrug resistance; *MDR-1* multidrug resistance protein 1 (P-gp, ABCB1); *MRP-1* multidrug resistance-associated protein 1 (ABCC1); *RAGE* the receptor for advanced glycation end products; *ROS* reactive oxygen species

### Nanotechnologic approch to facilitate flavonoid-conducted chemotherapeutic anti-cancer toxicity

Although flavonoids demonstrate significant anti-cancer and chemosensitizing efficacy in preclinical research, their poor solubility and bioavailability are associated with lesser effectiveness in vivo. However, as discussed below, current research highlights the potential enhancement of flavonoid-chemotherapy interaction through nanotechnology. Khonkarn et al. demonstrated that polymeric micelles of benzoylated methoxy-poly (ethylene glycol)-b-oligo(ε-caprolactone) or mPEG-b-OCL-Bz loaded with quercetin could represent an attractive tool to overcome MDR in cancer cells. Eventually, the combination of polymeric micelles (inhibiting P-gp efflux) and quercetin (interfering with the mitochondrial membrane potential) may represent crucial factors for the reversal of MDR in K562/ADR cells; quercetin also enhanced the cytotoxicity of doxorubicin and daunorubicin [116]. Also, the co-encapsulation of paclitaxel and naringin in mixed polymeric micelles improved the intracellular uptake and in vitro cytotoxicity of paclitaxel against breast cancer cells [117]. Further, the double-targeted nanocarrier, Quercetin-3′3-dithiodipropionic acid-Astragalus polysaccharides-Folic acid (QDAF), was synthesized and self-assembled into a neoteric nano-targeted delivery strategy, named nano-pomegranates, to effectively suppress MDR in estrogen receptor α (ERα)-positive breast cancer. Indeed, nano-pomegranates enhanced cellular uptake, apoptosis, and necrosis in MCF-7 cells in vitro and showed improved anti-cancer efficacy and lower systemic toxicity in vivo [118].

In conclusion, the co-delivery of conventional chemotherapeutic agents with flavonoids in nanocarrier systems could improve chemotherapeutics’ efficacy and enhance the chemosensitivity of cancer cells, inhibit chemoresistance, and reduce the cytotoxicity of chemotherapeutics in healthy tissues.

## Resistance to anti-cancer treatments

**Fig. 4:**
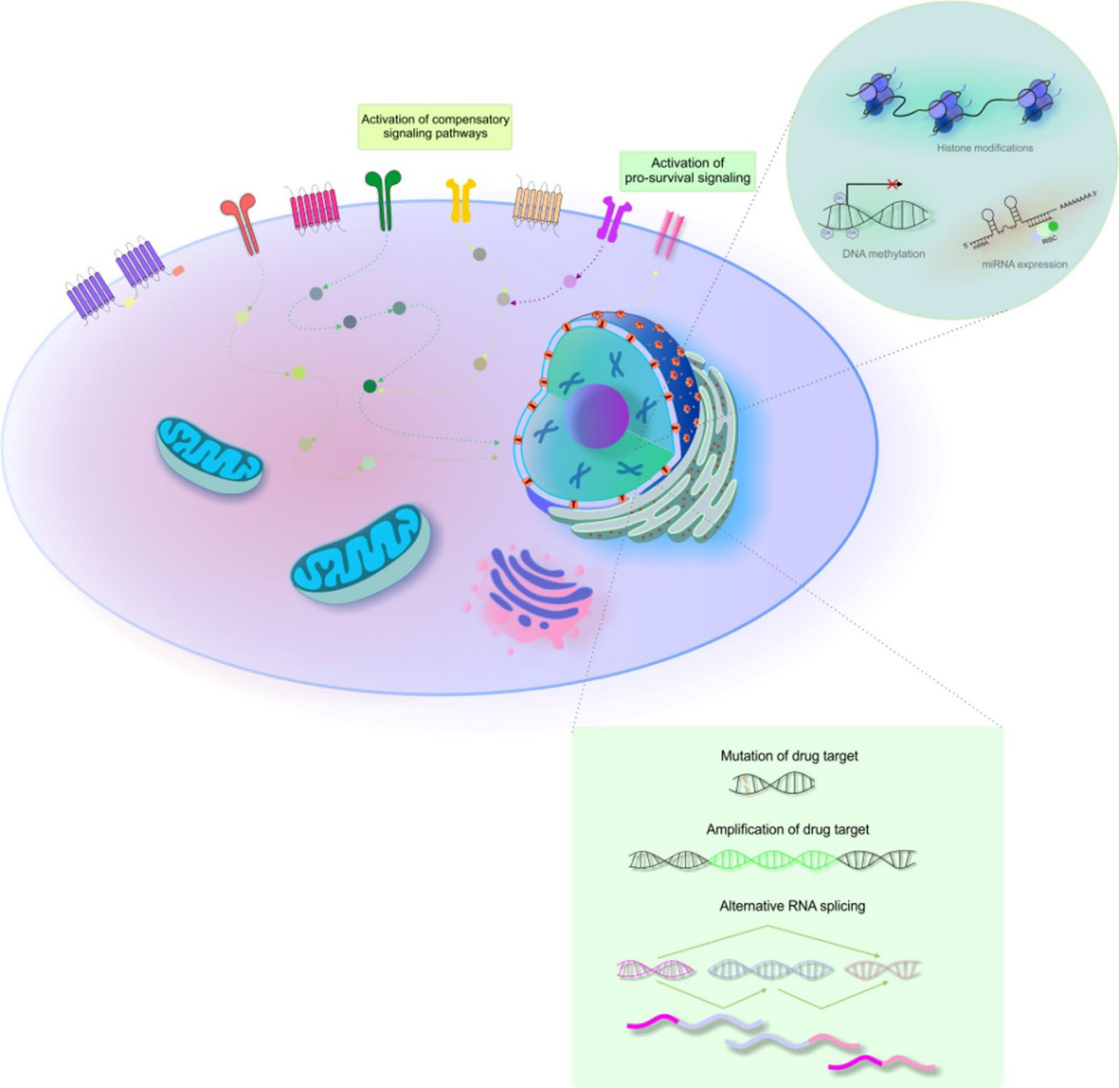
Mechanisms of cancer cells resistance to targeted therapy**. **Explanatory notes: Resistance to targeted therapy often results from reactivation of pathways inhibited by the drug (acquisition of drug-resistant mutations/amplification of the target, re-activation of downstream signalling proteins via activation mechanisms or activating mutations, or activation of compensatory signalling pathways) [123]. Due to commonly observed gene mutations, cancer cells can perform modifications as a response to targeted molecules and thus induce resistance to specific agents [12]. The mutation, amplification, downregulation, and alternative RNA splicing of drug targets all contribute to the resistance of cancer cells to targeted therapy [124]. Moreover, direct restoration of biologic function that was disrupted by a drug [125], activation of compensatory pathways parallel to or downstream of the inhibited pathway (such as pro-angiogenic signalling through PDGFR), activation of pro-survival signalling, and epigenetic alterations (like DNA methylation, histone modifications, and microRNA) also contribute to resistance to targeted treatment [124]

Conventional chemotherapy often fails due to resistance. Therefore, it was necessary to develop new strategies to improve the individual therapeutic efficacy of specific cancer types [119]. Molecular biology offers ideas for the development of selective drugs specifically targeted against certain tumors [9]. Similar to traditional chemotherapy, targeted anti-cancer agents modulate specific cellular processes (such as growth inhibition, apoptotic induction, and metastatic restriction). Unlike traditional chemotherapy, targeted cancer therapy also targets unique molecular changes associated with specific cancer types [14]. Thus, targeted cancer therapies focus on mutant proteins and signalling pathways essential for cancer cell survival and progression [120]. The term “targeted therapy” describes all treatment approaches affecting specific molecular targets and involves small selective inhibitory molecules and biological drugs such as monoclonal antibodies targeted against specific cellular receptors and proteins of neoplastic processes. Examples of monoclonal antibodies include bevacizumab, cetuximab, pertuzumab, and trastuzumab [9]. The targets of selective tyrosine kinase and serine/threonine-protein kinase small molecule inhibitors include growth factors, cell-cycle proteins, apoptotic modulators, signalling molecules, and molecules promoting angiogenesis. Imatinib, dasatinib, and nilotinib are selective tyrosine kinase inhibitors (TKIs). Small molecules targeting tyrosine kinase proteins include gefitinib and erlotinib. Further, lapatinib is a potent HER1/2 inhibitor. VEGF inhibitors are another class of TKIs, including sunitinib and sorafenib. Another class of selective small molecules includes mTOR inhibitors (temsirolimus and everolimus), BRAF inhibitors (vemurafenib and dabrafenib), MEK inhibitors (trametinib and cobimetinib), and inhibitors of proteasome machinery (bortezomib) [9]. In addition, abivertinib is a novel third generation epidermal growth factor receptor (EGFR) TKI [121] that inhibits Bruton’s tyrosine kinase (BTK), which exerts an oncogenic role in the proliferation and survival of many B cell malignancies [122]. Figure 4 provides an overview of mechanisms related to the resistance of cancer cells to targeted anti-cancer agents.

The resistance to single-agent targeted therapy is related to the occurrence of many cancer mutations, making such tumors less dependent on a single oncogenic event and more reliant on dynamic interconnected signalling pathways and tumor heterogeneity, especially in an advanced and metastatic stage [126]. Compared with the mode of resistance to cytotoxic agents associated with deregulated pharmacokinetics such as drug efflux, resistance to targeted therapy is usually a result of target gene mutations or the activation of pro-survival signaling. Therefore, combination therapy with next-generation agents, such as flavonoids, could target mutations and pathways associated with resistance as part of a personalized approach to mitigate targeted drug resistance in cancer patients [127].

### Flavonoids enhance effectiveness of targeted anti-cancer therapy

Targeted therapy tremendously enhances cancer management; however, acquired and intrinsic resistance are major limitations of targeted anti-cancer treatment [126]. Nevertheless, as discussed below, current research highlights the potential importance of flavonoids in increasing the sensitivity to and/or efficacy of current targeted anti-cancer agents.

Trastuzumab is a recombinant humanized monoclonal antibody targeted against the human epidermal growth factor receptor 2 (HER2) tyrosine kinase receptor and is used for the treatment of HER2-positive breast cancer. The anthocyanins cyanidin-3-glucoside and peonidin-3-glucoside inhibited trastuzumab-resistant breast cancer MDA‑MB‑453R and BT474R cells in vitro and in a murine xenograft model in vivo [128].

TKIs are novel target-specific anti-cancer drugs. Nevertheless, the disadvantage of TKIs usage is the development of resistance [54]. The existence of EGFR mutations in NSCLC led to changes in the traditional lung cancer regimen from traditional cytotoxic chemotherapy to molecularly targeted agents. Superior to traditional chemotherapy, EGFR TKIs are considered a standard first-line treatment modality for advanced NSCLC [129]. However, patients receiving EGFR TKIs usually develop resistance. Hydroxygenkwanin HGK, a novel flavonoid, exerted potent antitumor activity against TKI-resistant NSCLC cells by promoting the degradation of EGFR [55]. Similarly, the combination of apigenin and gefitinib [56], an orally active anilinoquinazoline that selectively and reversibly inhibits intracellular EGFR TKIs activity [130], could represent a strategy for acquired resistance to EGFR-TKIs in NSCLS as it blocked autophagy flux and induced apoptosis in lung cancer EGFR L858R-T790M-mutated H1975 cells [56]. The flavone apigenin synergized with abivertinib [122], a novel third-generation EGFR TKI [121] targeting BTK, inhibits diffuse large B-cell lymphoma in vitro (U2932, LY10, OCI-LY10 cells) and in a murine xenograft model through the inhibition of p-GS3K-β and its downstream targets; therefore, the ability of apigenin to synergize with BTK inhibitors is important for the improvement of targeted therapy, especially to overcome developed resistance [122]. Moreover, resistance mediated by BCR-ABL limits the utilization of TKIs in leukemia. Nevertheless, the chalcone xanthohumol attenuated the autophagy induced by imatinib, a small molecule TKI used to treat chronic myelogenous leukemia and enhanced its therapeutic efficacy in myelogenous leukemia K562 cells [111]. Further, *Trifolium* flavonoids showed a capacity to overcome resistance to gefitinib, EGFR-TKI, through suppressing ERK and STAT3 signaling in NSCLC cell line PC-9R [131]. A novel EGCG (flavonol) derivative isolated from Anhua dark tea sensitized NSCLC gefitinib-resistant cells HCC827-Gef to gefitinib through the suppression of PI3K/mTOR signalling and EMT [132].

Sorafenib is a multikinase angiogenesis inhibitor [9] used as first-line therapy in HCC. However, patients who initially benefit from sorafenib usually develop resistance within 6 months [133]. A proposed mechanism of this resistance is the expression of the pregnane X receptor (PXR) or MDR-1, which is related to the elimination of sorafenib in HCC cells. Interestingly, the flavone rhamnetin is an inhibitor of sirtuin (Sirt) 1 and could inhibit the downstream (PXR) gene—MDR-1, via the miR-148a/PXR axis. Therefore, rhamentin decelerated the metabolic clearance of sorafenib and also sensitized HCC cells to the drug [134]. Similarly, the combinatory treatment with apigenin potentiated the cytotoxicity of sorafenib in HCC HepG2 cells, as demonstrated through decreased cell viability, decreased migration and invasion, and increased apoptosis compared with single treatment groups [135]. Also, Saraswati et al. demonstrated the capability of a chalcone phloretin to overcome sorafenib resistance in HCC as demonstrated through Src homology region 2 domain-containing phosphatase-1 (SHP-1)-mediated inhibition of STAT3 and Akt/VEGFR2 [136]. As stated above, the disadvantage of TKIs usage is resistance; important mechanisms of the development of resistance include enhanced TKI efflux through efflux transporters such as BCRP [54]. 5,7-dimethoxyflavone effectively inhibited BCRP-1-mediated sorafenib efflux in Madin-Darby Canine Kidney Type II wild-type cell subclones that were transfected with murine Bcrp1 (MDCK/Bcrp1); these results highlight an essential potential of 5,7-dimethoxyflavone as a chemosensitizing agent in BCRP-mediated drug resistance [54]. Further, the flavonol kaempferol enhanced the chemotherapeutic efficacy of sorafenib against HCC demonstrated in silico and in vitro (liver cancer HepG2 and N1S1 cells); also, kaempferol reversed MDR by decreasing P-gp overexpression [137].

Moreover, the flavonoid derivative WYC0209 is a potential adjuvant agent against CD133-driven urothelial carcinoma (UC) CSCs and could serve as a potent strategy against UC therapeutic resistance; among others, WYC0209 declined EMT-CSCs markers such as MDR-1 or ABCG2 in vitro [138].

Furthermore, the flavone scutellarin potentiated the activity of bortezomib (a proteasome inhibitor), circumvented chemoresistance, promoted apoptosis, and repressed tumor growth in a murine xenograft model of multiple myeloma through the HDAC/miR-34a-mediated down-modulation of Akt/mTOR and NF-κB signaling [139].

Also, histone deacetylase inhibitors (HDACi) and tumor necrosis factor‑associated apoptosis‑inducing ligand (TRAIL) represent other targeted anti-cancer therapeutic strategies [140, 141]. HDACi is a novel class of small-molecular therapeutics that target the regulation of histone and non-histone proteins [142]. The flavonol fisetin is a potential complementary agent in HDACi resistance, as it improves the chemosensitivity of HA22T, apicidin-resistant, and suberoylanilide hydroxamic acid-resistant (SAHA-R) HCC cells. Fisetin synergistically interacted with HDACi in parental cells and also resistant cell lines. Fisetin also promoted therapeutic potential in the xenograft model generated from HDAC inhibitor-resistant cells [141]. Further, TRAIL is an immune cytokine of the TNF family that received attention as a targeted anti-cancer agent through the selective induction of apoptosis in cancer cells [143, 144]. Despite tumor TRAIL’s potential as a potent anti-cancer agent inducing apoptosis of cancer but not normal cells, colon cancer is often TRAIL-resistant. Mutations in DR4 and DR5, domains of death receptors associated with TRAIL-induced apoptosis, induce cancer cell resistance to TRAIL. However, icariin (a prenylated flavonol glycoside derived from *Epimedium sagittatum*) sensitized HCT116 colon cancer cells to TRAIL-induced apoptosis through the upregulation of DR5 and DR4 (mediated by ROS, ERK, and transcription factor CCAAT enhancer‑binding protein homologous protein/CHOP/) in vitro and in vivo (xenograft mouse model) [140].

Table 3 provides a summary of the mechanisms through which flavonoids enhance the therapeutic efficacy of targeted anti-cancer agents.

**Table 3:** Flavonoids enhancing the efficacy of targeted anti-cancer agents: Up-to-date evidence

Flavonoid	Targeted anti-cancer agent	Study details	Effects (mechanisms)	Reference
Anthocyanins (cyanidin-3-glucoside and peonidin-3-glucoside)	Trastuzumab	Trastuzumab‑resistant MDA‑MB‑453R and BT474R cell in vitro and murine xenograft model (BT474R cells implanted into nude mice)	Inhibition of resistance to trastuzumab (inhibition of HER2 phosphorylation, apoptotic induction, suppression of migration and invasion, and tumor cell growth inhibition)	[128]
Hydroxygenkwanin	TKI	TKI-resistant NSCLC cells	Targeting TKI-resistant cancer cells (EGFR degradation)	[55]
Apigenin	Gefitinib	EGFR L858R-T790M-mutated H1975 lung cancer cells	Combating resistance to EGFR-TKI (blocked autophagy flux and induced apoptosis)	[56]
Apigenin	Abivertinib	Diffuse large B-cell lymphoma (U2932, LY10, OCI-LY10) cells and xenograft model (U2932 cells injected into Balb/c male mice)	Apigenin synergizing with BTK inhibitors → improvement of targeted therapy to overcome developed resistance (inhibition of p-GS3K-β and its downstream targets; downregulation of PI3K)	[122]
Xanthohumol	Imatinib	Myelogenous leukemia (K562) cells	Enhanced efficacy of imatinib	[111]
*Trifolium* flavonoids	Gefitinib	NSCLC PC-9R cells	Overcome resistance to gefitinib (suppressing ERK and STAT3 signalling)	[131]
EGCG derivative	Gefitinib	NSCLC gefitinib-resistant cells HCC827-Gef	Sensitized resistant cells to gefitinib (suppression of PI3K/mTOR signalling and EMT)	[132]
Rhamnetin	Sorafenib	HCC cells	Promoted sensitivity of HCC cells to sorafenib (decreased expression of PXR or MDR-1 associated with the elimination of sorafenib in HCC cells via the miR-148a/PXR axis)	[134]
Apigenin	Sorafenib	HCC HepG2 cells	Potentiated the cytotoxicity of sorafenib in HCC (decreased cell viability and migration and invasion; increased apoptosis)	[135]
Phloretin	Sorafenib	HCC	Overcame sorafenib resistance in HCC cells (SHP-1-mediated inhibition of STAT3 and Akt/VEGFR2)	[136]
5,7-dimethoxyflavone	Sorafenib	MDCK/Bcrp1 cells	Chemosensitizing BCRP-mediated drug resistance (inhibited sorafenib efflux mediated by BCRP-1)	[54]
Kaempferol	Sorafenib	In silico and in vitro (liver cancer HepG2 and N1S1 cells)	Enhanced chemotherapeutic efficacy of sorafenib against HCC; reversed MDR by decreasing P-gp overexpression	[137]
WYC0209	UC therapeutic resistance	T24, BFTC905 and BFTC909 UC cell lines	Potential as an adjuvant agent against CD133-driven urothelial carcinoma CSCs and a strategy against UC therapeutic resistance (decreased EMT-CSCs markers such as MDR-1 or ABCG2)	[138]
Scutellarin		Multiple myeloma murine xenograft model (Balb/c mice injected with MM.1S cells)	The potentiated activity of bortezomib, circumvented chemoresistance (HDAC/miR-34a-mediated down-regulation of Akt/mTOR and NF-κB signalling)	[139]
Fisetin		HCC cells (HA22T, apicidin-R, and suberoylanilide hydroxamic acid resistant (SAHA-R) cells and xenograft model generated from HDAC inhibitor-resistant cells (HA22T and HDACis-R cells in nude mice)	Potential as a complementary agent in HDACi resistance (improving the chemosensitivity of HCC cells)	[141]
Icariin		Colon cancer HCT116 cells in vitro and murine xenograft model	Sensitization of cancer cells to TRAIL-induced apoptosis (upregulation of DR5 and DR4 mediated by ROS, ERK, and CHOP)	[140]

*ABCG2* breast cancer resistance protein (BCRP); *BCRP-1* Breast Cancer Resistance Protein; *BTK* Bruton’s tyrosine kinase; *CSC* cancer stem cells; *EGFR* epidermal growth factor receptor; *EMT* epithelial-mesenchymal transition; *HCC* hepatocellular carcinoma; *HDACi* histone deacetylase inhibitors; *HDACis-R* histone deacetylase inhibitors-resistant; *HER2* human epidermal growth factor receptor 2; *MDCK-II/Bcrp1* Madin-Darby Canine Kidney Type II (MDCK-II) wild type cells subclone transfected with murine Bcrp1; *MDR* multidrug resistance; *MDR-1* multidrug resistance protein 1; *NSCLC* non-small cell lung cancer; *PXR* pregnane X receptor; *ROS* reactive oxygen species; *SHP-1* Src homology region 2 domain-containing phosphatase-1; *TKI* tyrosine kinase inhibitors; *UC* urothelial carcinoma

### Nanotechnological and combinatorial approaches enhance effectiveness of the flavonoids-conducted therapy 

Combinatorial and nanoparticulate approaches are suggested to overcome the challenges of resistance and severe side effects posed by monotherapies. Currently, the combinatory therapy of a chemotherapeutic agent and phytochemicals or chemotherapy and targeted therapy is an important tool to improved cancer patient management. Chemotherapy combined with targeted therapy is suggested to be effective especially for advanced NSCLC while EGFR is an essential target in NSCLC patients. Cetuximab, a monoclonal antibody targeting EGFR, is a first-line treatment for NSCLC, advanced colorectal cancer, and head and neck cancers. Indeed, cetuximab-functionalized nanostructured lipid carriers were developed for the co-delivery of paclitaxel and 5-demethylnobiletin (a hydroxylated polymethoxyflavone from citrus) and to avert dose-related adverse effects of anti-cancer agents. These nanostructured lipid carriers effectively inhibited tumor growth in a model of A549 paclitaxel-resistant cell-bearing mice [145].

In conclusion, flavonoids represent an effective tool to improve the therapeutic outcomes of targeted anti-cancer strategies facing evident disadvantages such as insensitivity and resistance.

## Anti-cancer immunotherapy

### Imuunotherapy revolutionized treatments of malignancies

After 2010, cancer immunotherapy research introduced new monoclonal antibodies targeting tumor antigens and T-cell protein receptors to downregulate the immune response, specifically the immune checkpoint inhibitor monoclonal antibodies anti-cytotoxic T-lymphocyte-associated antigen 4 (anti-CTLA4) and anti-programmed cell death protein 1 antibody (anti-PD1). Monoclonal antibodies directed against immune checkpoint inhibitors include ipilimumab, nivolumab, and pembrolizumab [9]. Immunotherapy is a promising tool for cancer management, as it restores the anti-tumor immune response [15]. Not all patients respond to immunotherapy; thus, it is necessary to improve its efficacy [15].

Some level of immune escape and resistance is intrinsic to malignancies due to the development of most human tumors in an immune-competent environment. However, acquired resistance to immunotherapy can result from pre-existing genetic and epigenetic traits or de novo alterations of cancer cells or other tumor microenvironmental components. Thus, cancer cells can evade the immune response intrinsically (loss or downregulation of target antigen expression, defective antigen presentation, insensitivity to immune effector molecules, upregulation of alternative immune checkpoints, and epigenetic alterations) or via extrinsic mechanisms, which are mediated by non-cancer cells of the tumor microenvironment including tumor-associated macrophages (TAMs), regulatory T cells (Tregs), and myeloid-derived suppressor cells (MDSCs) [146].

Programmed death ligand 1 (PD-L1) is an essential immune checkpoint protein that binds to programmed death 1 (PD-1) on T lymphocytes. Indeed, T cells exert an essential role in the eradication of cancer cells. However, cancer cells escape the immune response through PD-L1 expression. The binding of PD-L1 to PD-1 results in the inhibition of T-cell proliferation and activity, leading to tumor immunosuppression [147]. Although PD-L1/PD-1 checkpoint inhibition revolutionized the treatment of various malignancies, such therapy is still ineffective in a significant percentage of patients due to primary or acquired resistance [148].

Due to the ineffectiveness of immunotherapy and the experience of resistance in some cases, the antitumor efficacy of cancer immunotherapy needs to be increased. Thus, immunotherapeutic agents are often administered in combination with each other or with chemotherapeutic agents, radiotherapy, or surgery. Also, the combination of immunotherapy with antiangiogenic drugs yields promising outcomes [9, 149]. It is also essential to emphasize the potential of phytochemicals and their derivatives to improve cancer immunotherapy responses in the development of novel immunotherapeutic strategies [150, 151].

### Flavonoids enhance effectiveness of anti-cancer immunotherapy

As discussed below, the anti-cancer effects of flavonoids are also applicable in cancer immunotherapy either in combination with other agents or single agents [57, 59, 152–155].

Due to the frequent development of resistance to sorafenib, the first-line therapy for HCC, immune checkpoint inhibitors (ICI) such as nivolumab are studied as alternatives. However, due to the often unsuccessful outcomes of immunotherapy, the combinatorial approach seems to be a better choice to improve the treatment and to block immunosuppressive signals in the tumor microenvironment. Although the co-administration of VEGF inhibitors and ICI is associated with synergistic anti-cancer effects, it exerts several adverse effects. However, phytochemicals including flavonoids could improve the plant-based antiangiogenic-immunotherapy combination in HCC when compared with single compounds that are often associated with therapeutic failure [152].

Furthermore, flavopiridol is a synthetic flavonoid that inhibits cyclin-dependent kinases [156]. Although most chronic lymphocytic leukemia (CLL) patients receiving chemoimmunotherapy achieve complete remission, patients with significantly shortened progression-free intervals still represent an important obstacle. Also, minimal residual disease (MRD) occurs in a majority of CLL patients who relapse. Moreover, a phase I clinical trial demonstrated flavopiridol to be safe and efficient as consolidation therapy after chemoimmunotherapy in CLL patients [153].

As discussed above, the immune escape of cancer cells is associated with PD-L1 expression [147]. Also, the chemoresistance of nasopharyngeal carcinoma is associated with the upregulation of checkpoint inhibitor PD-L1, which is linked to enhanced aerobic glycolysis promoted by HIF1-α deregulation and LDH-A activity. However, silibinin downregulated PD-L1 expression by modulating HIF-1α/LDH-A-mediated metabolism in nasopharyngeal carcinoma C666-1 cells and thus provided a potential avenue to overcome PD-L1-mediated resistance [57].

Moreover, checkpoint blockade is an effective treatment of lung cancer; however, it often leads to resistance. Therefore, Tang et al. aimed to develop a new strategy to improve checkpoint blockade therapy. Eventually, dual inhibition of COX-2 and EGFR by melafolone improved PD-1 immunotherapy against Lewis lung carcinoma and CMT167 tumors; these results highlight its important role as a combinatory strategy against lung cancer by affecting vessels and immune cells [59]. Further, the prenylated flavonoid icaritin exerts potent anti-cancer activity by modulating multiple biochemical and cellular responses [58]. Advanced HCC is associated with limited treatment options. It is suggested that icaritin has the potential as an oral immunotherapeutic agent used alongside immune-checkpoint inhibitors (antibody-based PD-1/PD-L1 blockade therapies). As the authors demonstrated in a phase I trial, the preliminary durable survival benefits of icaritin in advanced HCC patients correlated with its immuno-modulatory activities and immune biomarkers [154]. Similarly, apigenin also suppressed PD-L1 in vitro in melanoma cells and in host dendritic cells; this potentiated the cytotoxicity of cocultured cytokine-induced killer cells against melanoma cells [155].

In conclusion, flavonoids improve cancer immunotherapeutic effects either through increased efficacy of other anti-cancer agents or as potent single molecules modulating immune responses of cancer cells.

## Conclusions and expert recommendations in context of predictive, preventive and personalized (3P) medicine

Cost-efficacy of currently applied treatments is an issue in overall cancer management challenging healthcare and causing tremendous economic burden to societies around the world. Consequently, complex treatment models presenting concepts of predictive diagnostics followed by the targeted prevention and treatments tailored to the individualized patient profiles earn global appreciation as benefitting the patient, healthcare economy, and the society at large.

In this context, application of flavonoids as a spectrum of compounds and their nano-technologically created derivatives is extensively under consideration, due to their multi-faceted anti-cancer effects applicable to the overall cost-effective cancer management, primary, secondary, and tertiary prevention.

Conventional anti-cancer strategies demonstrate evident deficits. Despite recent progress in anti-cancer strategies, the development of a therapy resistance remains the leading cause of the cancer-related mortality. An improved understanding of carcinogenic processes allows for the technological innovation creating more efficient therapeutic modalities. Targeted anti-cancer therapies leverage unique molecular changes associated with specific cancer types.

Anti-cancer protective application of flavonoids in the context of 3P medicine should follow principles of the evidence-based therapeutic effects, individualized prediction, targeted prevention and personalization of the treatment algorithms. To this end, application of specialized analytical approaches is strongly recommended such as liquid biopsy analysis, risk assessment tools, predictive and companion diagnostics, multi-omics and multi-parametric analysis, and application of artificial intelligence in medicine.

## Data Availability

Not applicable.
